# Individual characteristics associated with youth symptom reports and persisting symptoms after concussion

**DOI:** 10.1111/jnp.70031

**Published:** 2026-01-31

**Authors:** S. D. Hicks, B. A. Harding, R. Olympia, J. Loeffert, C. Onks, R. C. Mannix, D. A. Levine, K. O. Yeates

**Affiliations:** ^1^ Department of Pediatrics, Penn State College of Medicine Penn State University Hershey Pennsylvania USA; ^2^ Department of Emergency Medicine, Penn State College of Medicine Penn State University Hershey Pennsylvania USA; ^3^ Department of Family and Community Medicine and Orthopedics and Rehabilitation, Penn State College of Medicine Penn State University Hershey Pennsylvania USA; ^4^ Division of Emergency Medicine Boston Children's Hospital, Harvard Medical School Boston Massachusetts USA; ^5^ Department of Emergency Medicine and Pediatrics, Weill Cornell Medicine NewYork Presbyterian Hospital New York New York USA; ^6^ Department of Psychology, Pediatrics, and Clinical Neurosciences University of Calgary Calgary Alberta Canada

**Keywords:** head impact, mild traumatic brain injury, neuropsychiatric, paediatrics, PSaC, sports

## Abstract

Dissonant approaches for measuring persisting symptoms after concussion (PSaC) make it difficult to predict who will experience prolonged symptoms. We sought to identify medical and sociodemographic characteristics associated with symptom burden and assess how such factors shape symptom evolution and PSaC classification after mild traumatic brain injury (mTBI). This analysis involved 1947 Post‐Concussion Symptom Scale reports from 1117 youths (11–21 years): 380 with mTBI, 737 without mTBI (194 healthy non‐mTBI; 543 non‐mTBI with underlying medical conditions). Multivariate regressions were used to assess the relationship of symptom burden with medical and sociodemographic factors among non‐mTBI youths, and interrogate how these factors impacted longitudinal symptom burden among mTBI participants. PSaC rates were characterized in the mTBI group 30 days after injury using 5 definitions: symptom burden, symptom severity, simple change in symptom burden and severity, and response to, ‘What percent of normal do you feel?’ In the absence of mTBI (i.e. baseline), symptom burden was associated with female sex, neuropsychiatric history, BMI and orthopaedic injury. In the 30 days following mTBI, age, sex and neuropsychiatric history were associated with symptom burden. Smaller household size, sports participation and parent education were protective. Rates of PSaC were 14.7–18.9%, displaying 84% agreement across the 5 definitions. However, PSaC misclassification was high among non‐mTBI youths with underlying medical conditions (37.2–50.6%), especially with the single‐item screener. Medical and sociodemographic factors affect concussion symptom reports and influence PSaC rates. A single‐item screener for PSaC may be useful but risks over‐detection among certain youths.

## INTRODUCTION

Identifying the subset of individuals who experience persisting symptoms after mild traumatic brain injury (mTBI) is challenging (Lumba‐Brown et al., 2018). Several factors contribute to this clinical challenge, including the subjective nature of symptom reporting, the wide range of concussion‐related symptoms that overlap with other medical conditions, and the lack of consensus for defining persisting symptoms (Makdissi et al., 2017). This clinical dilemma is also reflected in evolving terminology, from ‘post‐concussion syndrome’, to ‘persistent post‐concussion symptoms’, and most recently, ‘persisting symptoms after concussion’ (PSaC) (Broshek et al., 2022; Yeates et al., 2023; Young, 2020).

Understanding the incidence of PSaC is critical for accurate prognoses and diagnoses (Babikian et al., 2011; Barlow et al., 2015; Yeates et al., 2009). A meta‐analysis has estimated the prevalence of PSaC among youths to be 35.1%, but other studies have indicated prevalence may be as low as 11.8% to 16.1% (Barlow et al., 2015; Cancelliere et al., 2023; Chadwick et al., 2022). Variability in defining PSaC contributes to confusion about its true incidence (Voormolen et al., 2018). For example, some studies define PSaC as symptoms lasting beyond 2 weeks, while others use a more conservative definition of 3 months (Babcock et al., 2013; Eisenberg et al., 2014; Howell et al., 2016; Rose et al., 2015). Since the proportion of symptomatic individuals shrinks over time, PSaC rates are inextricably linked to symptom duration (Eagle et al., 2020; Eisenberg et al., 2014; Ledoux et al., 2019).

Heterogeneity also exists among standardized symptom measures (Alla et al., 2009; Elbin et al., 2016). Many studies use ICD‐10 criteria to derive a symptom threshold for PSaC based on the number of persisting symptoms (i.e. symptom burden) or the severity of persisting symptoms (i.e. symptom severity) (Eagle et al., 2020; Iverson et al., 2021; Mayer et al., 2020; Yeates et al., 2023). However, this approach tends to increase the likelihood of false positives. In addition, burden and severity thresholds vary across different symptom questionnaires because of differences in the number and content of items, as well as their response scales (e.g. 4 vs. 6‐point Likert) (Karaliute et al., 2021; Zeldovich et al., 2022). Sociodemographic factors, including age and sex, may also influence the extent to which individuals report concussion symptoms (Brown et al., 2015; Chadwick et al., 2022; Custer et al., 2016; Mayer et al., 2024; Moser et al., 2019). For example, a study of 31,958 high school athletes from Maine found that female sex was associated with higher symptom burden, and 28% of non‐concussed girls had a symptom burden resembling PSaC (Iverson et al., 2015). Symptom burden in this cohort was also influenced by medical conditions, including prior mTBI, migraine history and ADHD. Such studies are critical for understanding the factors that influence concussion symptom reporting, yet most rely on baseline reports from college or high school athletes (Asken et al., 2017; Iverson et al., 2015; Piland et al., 2010).

To address the limitations associated with variation across individual symptom reporting, standardized change in symptoms can be assessed through ratings of symptoms before injury (pre‐injury or retrospective) (Black et al., 2020; Teel et al., 2019). Yet, even this approach has limitations, particularly in paediatric populations (Mayer et al., 2020). Correspondence between self‐report and parent report is generally modest, and individuals may over‐ or under‐estimate retrospective ratings (Gunstad & Suhr, 2001; Mayer et al., 2020). A recent systematic review determined that standardized symptom scales remain the most reliable way to detect PSaC (Yeates et al., 2023). However, divergent criteria continue to result in differing estimates of PSaC incidence, hampering advances in concussion care, such as large therapeutic trials (Mayer et al., 2020; Yeates et al., 2023).

The purpose of this study was to identify sociodemographic and medical correlates of PSaC among youths, especially those with pre‐existing mental health conditions and non‐athletes, who have been historically under‐represented in concussion research. Such knowledge could enhance approaches to PSaC classification and improve concussion care. The study's goals were to: (1) assess the impact of medical and demographic characteristics on youths' concussion symptom reports in the absence and presence of mTBI; and (2) examine how various PSaC classification approaches affect PSaC rates. To achieve these goals, we first used the Post‐Concussion Symptom Scale (PCSS) to characterize concussion symptoms in healthy youths without mTBI and establish normative cut‐offs for PSaC (Sady et al., 2014). Next, we examined concussion symptoms among both healthy youths without mTBI and youths without mTBI whose underlying medical conditions mimic concussion‐like symptoms. Finally, we assessed rates of PSaC in a cohort of youths with mTBI, determining how medical and sociodemographic characteristics influenced longitudinal symptom reports and ultimate PSaC classification.

## METHODS

### Research ethics approval

This study received research ethics approvals from the Institutional Review Board (IRB) at the Pennsylvania State College of Medicine (STUDY00014022 and STUDY00003729), as well as multi‐site approval from the Western IRB (#1271583). Informed, written consent was obtained for all participants and informed assent was obtained where appropriate.

### Participants

This study involved a retrospective analysis of data from two longitudinal, multi‐centre cohort studies that enrolled participants between August 2017 and September 2023 (Figure S1). Participants included youths with a clinical diagnosis of mTBI (*n* = 380), as well as youths without mTBI (*n* = 737). For all participants, inclusion criteria were age 11–21 years, English language fluency and parent/guardian ≥18 years of age. Additional inclusion criteria for youths with mTBI were clinical diagnosis of mTBI (based on the 2016 Berlin consensus statement on concussion in sport) (McCrory et al., 2017), and clinical presentation within 14 days of initial mTBI. Exclusion criteria for youths with mTBI were penetrating head injury or skull fracture, drug/alcohol dependency, ongoing seizures or moderate/severe TBI. Youths without mTBI were excluded for unresolved symptoms from a prior TBI, ongoing seizures or drug/alcohol dependency. Youths without mTBI (*n* = 737) were sub‐divided into ‘healthy, non‐mTBI’ (*n* = 194) and ‘non‐mTBI with underlying medical conditions’ (*n* = 543). The latter group included medical conditions that can mimic mTBI symptomology (i.e. attention deficit hyperactivity disorder (ADHD), anxiety, chronic headaches, depression, learning disability and orthopaedic injury [OI]).

Enrolment took place at outpatient clinics, emergency departments and athletic training clinics affiliated with 11 institutions. Targeted enrolment at outpatient neurology clinics, orthopaedic clinics and psychiatric clinics was used to enrich the non‐mTBI group for conditions that can mimic mTBI symptomology, allowing us to examine their impacts on symptom reporting in the absence of mTBI. Presence of comorbid conditions was self‐reported and confirmed through medical record review for all participants without mTBI.

### Study definitions

The primary outcomes of the study were symptom burden, defined as the total number of symptoms rated >0 on the PCSS (Asken et al., 2017) and PSaC, defined by patient‐reported symptoms on the PCSS 30 days after injury (based on recommendations from the Berlin expert consensus panel and the ICD‐10) (McCrory et al., 2017). First, the association between medical and sociodemographic factors with symptom burden was assessed for youths without mTBI (both healthy non‐mTBI and non‐mTBI with an underlying medical condition) and for youths with mTBI. Then, rates of PSaC were compared across five PSaC classification approaches: (1) a symptom burden threshold (set by the 90th percentile symptom burden among healthy individuals without mTBI) (O'Brien et al., 2021); (2) a symptom severity threshold (set by the 90th percentile symptom severity among healthy individuals without mTBI); (3) an increase of ≥2 symptoms from self‐reported pre‐injury symptom burden (Hearps et al., 2017); (4) an increase of >3 points from self‐reported pre‐injury symptom severity (Barlow et al., 2010; Bressan et al., 2016); and (5) response to the single question, ‘What percent of normal do you currently feel, on a scale of 0–100?’ The first four approaches have been utilized in prior studies of PSaC (Barlow et al., 2010; Bressan et al., 2016; Hearps et al., 2017; O'Brien et al., 2021), whereas the fifth approach is novel.

### Measures

Electronic surveys were used to collect all data. Parents were permitted to assist children with survey completion. The PCSS, from the Sports Concussion Assessment Tool—5th Edition (SCAT‐5), was used to assess the severity of 22 concussion‐related symptoms on a 0–6 Likert scale, for all participants, regardless of mTBI status. Youths with mTBI were asked to repeat this assessment 1–2 weeks after enrolment, and again 30 days after their mTBI. They also retrospectively estimated their pre‐injury symptom level at the time of enrolment (i.e. 2.9 ± 2 days post‐injury). Scores on the PCSS were used to calculate symptom burden (i.e. the total number of symptoms rated >0; out of 22 possible symptoms) and symptom severity (i.e. the sum of all 22 Likert scores; out of 132 possible points).

At the time of enrolment, the following medical and demographic factors were also collected from all participants: age (years), sex, race, ethnicity, sports participation, body mass index (BMI; kg/m^2^), parental education, household size, history of prior concussion and presence/absence of six medical conditions (chronic headache, ADHD, depression, anxiety, LD and OI). Youths with mTBI reported whether these conditions existed prior to their injury. For youths with mTBI, the following injury‐related parameters were collected: mechanism of injury, presence/absence of immediate signs (i.e. amnesia, emesis, loss of consciousness and weakness), time since injury (days) and participation in mTBI‐specific therapy. Participants with missing PCSS data at enrolment (246/1363, 18%) were excluded from analysis (Figure S1). Among the 1117 participants with complete PCSS data, there were a total of 1660 missing responses for other variables (3.8% of all responses), most commonly regarding household size (363/1117, 32%) and learning disability (318/1117, 28%) (Table 1). A k‐nearest neighbour technique, well‐suited for both continuous and categorical variables, was used to impute missing values (Tavazzi et al., 2020). For downstream multivariate regression analyses, the primary outcome measure (symptom burden) was log‐transformed and pareto scaled to correct for non‐normality.

**TABLE 1:** Participant characteristics.

Characteristic, *n* (%)	All participants (*n* = 1117)	mTBI (*n* = 380)	All non‐mTBI (*n* = 737)	Healthy non‐mTBI (*n* = 194)	Non‐mTBI with underlying medical condition (*n* = 543)
Demographics					
Age, years (mean, SD)	16.1 (2.8)	16.4 (2.3)	15.9 (3.0)	17.5 (3.5)	15.3 (2.5)
Biologic sex, female	528 (47.3)	167 (43.9)	361 (49.0)	84 (43.2)	277 (51.0)
Race					
Asian	19 (1.7)	8 (2.1)	11 (1.5)	5 (2.6)	6 (1.1)
Black or African American	61 (5.5)	22 (5.8)	40 (5.4)	15 (7.7)	25 (4.6)
Bi‐racial	69 (6.2)	21 (5.5)	47 (6.4)	4 (2.1)	43 (7.9)
Hawaiian or Pacific Islander	1 (0.1)	0 (0)	1 (0.1)	0 (0.0)	1 (0.2)
Other	52 (4.7)	22 (5.8)	30 (4.1)	7 (3.6)	22 (4.0)
Prefer not to answer	126 (11.3)	93 (24.5)	33 (4.5)	22 (11.3)	11 (2.0)
White	789 (70.6)	214 (56.3)	575 (78.0)	141 (72.7)	434 (79.9)
Ethnicity					
Hispanic	94 (8.4)	33 (8.7)	61 (8.3)	12 (6.2)	49 (9.0)
Non‐Hispanic	796 (71.3)	194 (51.1)	602 (81.7)	159 (81.9)	443 (81.6)
Prefer not to answer	74 (6.6)	0 (0)	74 (10.0)	23 (11.9)	51 (9.4)
Unknown	153 (13.7)	153 (40.3)	0 (0)	0 (0)	0 (0)
Sports participation, yes	705 (69.5)	276 (86.8)	429 (61.6)	144 (81.8)	285 (54.8)
SDOH					
Parent Education					
≥ Bachelor's Degree	353 (31.6)	92 (24.2)	261 (35.4)	50 (25.8)	211 (38.9)
< Bachelor's Degree	364 (32.6)	82 (21.6)	282 (38.3)	33 (17.0)	249 (45.9)
Unknown	400 (35.8)	206 (54.2)	194 (26.3)	111 (57.2)	83 (15.2)
Household size, # of persons (median, IQR)	4 (2)	4 (1)	4 (2)	4 (1)	4 (2)
Medical characteristics					
BMI, kg/m^2^ (SD)	24.0 (5.9)	23.8 (5.5)	24.0 (6.1)	23.2 (3.4)	24.2 (6.7)
Prior TBI	255 (23.1)	111 (30.1)	144 (19.5)	24 (12.4)	120 (22.1)
ADHD	253 (31.2)	33 (16.3)	220 (36.2)	0 (0.0)	220 (42.4)
Anxiety	303 (37.8)	37 (18.9)	266 (43.9)	0 (0.0)	266 (51.4)
Chronic headaches	246 (30.3)	35 (16.7)	211 (35.0)	0 (0.0)	211 (41.0)
Depression	212 (26.3)	23 (11.5)	189 (31.2)	0 (0.0)	189 (36.6)
Learning disability	66 (8.3)	8 (4.1)	58 (9.6)	0 (0.0)	58 (11.2)
Orthopaedic injury	63 (7.9)	10 (5.1)	53 (8.8)	0 (0.0)	53 (10.3)
Sleep disorder	32 (5.5)	8 (4.3)	24 (6.1)	0 (0.0)	24 (6.4)
mTBI Characteristics					
Injury mechanism					
Fall		42 (11.6)			
Other		50 (13.8)			
Sport		252 (69.4)			
Immediate symptoms					
Amnesia		91 (32.6)			
Emesis		14 (7.4)			
LOC		62 (23.0)			
Weakness		72 (38.1)			
Time since injury (days)					
Enrolment, mean (SD)		2 (2)			
Follow‐up #1, mean (SD)		10 (3)			
Follow‐up #2, mean (SD)		31 (3)			
mTBI‐specific therapy, yes		6 (5.1)			

*Note*: Missing data for sports participation (103), household size (369), BMI (69), prior TBI (11), ADHD (307), anxiety (315), chronic headaches (304), depression (312), learning disability (318), orthopaedic injury (317). Abbreviations: Body mass index (BMI), attention deficit hyperactivity disorder (ADHD), mild traumatic brain injury (mTBI), motor vehicle accident (MVA), social determinants of health (SDOH).

### Statistical analyses

Descriptive statistics were used to report the characteristics of youths with mTBI (*n* = 380) and youths without mTBI (*n* = 737; divided into 194 healthy non‐mTBI and 543 non‐mTBI with an underlying medical condition). Among the non‐mTBI youths with an underlying medical condition (*n* = 543), a multivariate linear regression analysis was used to assess the association of symptom burden with 13 medical and sociodemographic factors (age, sex, BMI, household size, parent education, prior mTBI, sports participation, ADHD, anxiety, chronic headache, depression, learning disability and OI). Next, a mixed model fit by restricted maximum likelihood was used to assess how those same medical and sociodemographic factors impacted symptom burden over time among youths with mTBI (*n* = 380). Participant ID served as the clustering variable, and days‐since‐injury was used as a covariate. Kolmogorov–Smirnov tests, Goldfeld–Quandt tests and Durbin–Watson tests were used to check assumptions regarding normality, heteroskedasticity and autocorrelation, respectively. A power analysis demonstrated that the non‐mTBI youths with an underlying medical condition (*n* = 543) provided 80% power to detect an effect size of pη^2^ > 0.0143 in a multiple regression with 13 predictors and alpha set at 0.05 (Muller et al., 1992). The mTBI group (*n* = 380) provided 80% power to detect an effect size of pη^2^ > 0.0203 in a multiple regression employing 13 predictors with alpha set at 0.05. Since standard regression theory accounts for multiple predictors, particularly in a model that uses a single outcome and seeks to confirm previously described relationships using pre‐specified predictors, multiple testing correction was not employed. Finally, rates of PSaC were assessed for youths with mTBI who provided PCSS data at 30 days post‐injury (*n* = 258). Rates of PSaC were compared across the five PSaC definitions. Agreement was assessed using Fleiss' Kappa. PSaC misclassification rates among youths without mTBI were also reported.

## RESULTS

### Participant characteristics

This cohort study included 1117 youths, 11–21 years of age (Table 1). Nearly half of participants were female (47.3%). Most were White (70.6%), non‐Hispanic (71.3%) and participated in sports (69.5%). Nearly one quarter had a prior TBI (24.0%). Rates of ADHD (31.2%), anxiety (37.8%), chronic headaches (30.3%), depression (26.3%), learning disability (8.3%) and OI (7.9%) were relatively higher than the US population due to the targeted enrolment of youths with these conditions.

### mTBI characteristics

The most common mechanism of mTBI was sport‐related (69.4%) (Table 1). Weakness (38.1%) and amnesia (32.6%) were the most reported signs immediately following mTBI. Enrolment occurred, on average, 2 (±2) days after injury. Follow‐up assessments were completed, on average, 10 (±3) and 31 (±3) days after injury, respectively. Few participants (5.1%) received TBI‐specific therapy during the study period.

### Symptoms among healthy, non‐mTBI youths

**FIGURE 1:**
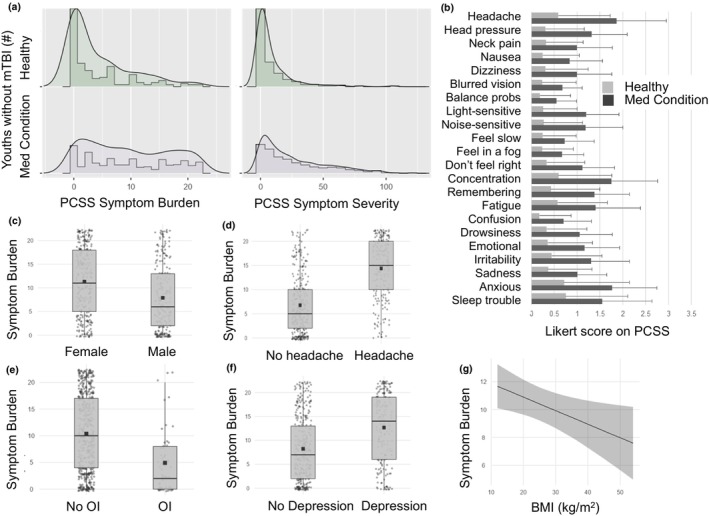
Symptom reports among youths without mild traumatic brain injury (mTBI) (healthy non‐mTBI and non‐mTBI with an underlying medical condition). The bar plots display symptom burden and symptom severity on the Post‐Concussion Symptom Scale (PCSS) for 737 youths without mTBI (194 healthy, non‐mTBI, 543 non‐mTBI with an underlying medical condition) (a). Mean Likert ratings (0–6) for each of the 22 items on the PCSS are also displayed for non‐mTBI sub‐groups (b). Violin plots display symptom burden across medical and demographic factors that were related to symptom burden among non‐mTBI youths with an underlying medical condition on multivariate regression analysis, including sex (c), chronic headaches (d), depression (e), orthopaedic injury (OI; f) and body mass index (BMI; g). The relationship between BMI and symptom burden is displayed via scatter plot, where shaded areas represent 95% confidence intervals.

Among healthy non‐mTBI youths (*n* = 194), the median symptom burden was 1 out of 22 possible symptoms (range: 0–22; IQR: 7.0; 90th percentile: 13) and the median symptom severity was 2 out of a possible 132 points (range: 0–104; IQR: 10; 90th percentile: 21) (Figure 1a). Mean Likert scores for healthy non‐mTBI youths were highest for trouble falling asleep (0.75, SEM = 0.09), nervous/anxious (0.71; SEM = 0.08), difficulty concentrating (0.60; SEM = 0.08) and headache (0.59; SEM = 0.08) (Figure 1b).

### Symptoms among non‐mTBI youths with an underlying medical condition

Among non‐mTBI youths with an underlying medical condition (*n* = 543), the median symptom burden was 9 out of 22 symptoms (range: 0–22; IQR: 13; 90th percentile: 20) and the median symptom severity was 17 out of 132 points (range: 0–121; IQR: 34; 90th percentile: 65) (Figure 1a). Mean Likert scores for non‐mTBI youths with an underlying medical condition were highest for headache (1.8; SEM = 0.08), difficulty concentrating (1.8; SEM = 0.08), nervous/anxious (1.7; SEM = 0.08) and trouble falling asleep (1.5; SEM = 0.08) (Figure 1b).

### Factors associated with symptom reports among non‐mTBI youths with an underlying medical condition

A multivariate regression employing 13 medical and sociodemographic factors (age, sex, BMI, parent education, household size, sport participation, prior mTBI, ADHD, anxiety, chronic headaches, depression, learning disability, OI) accounted for 35.5% of the variance in symptom reporting (RMSE = 5.74, AIC = 3348, *F* = 23.2, *p* < 0.001) (Table 2a). Female sex (Est = 1.27 [0.18–2.36]), chronic headaches (Est = 7.05 [5.93–8.17]) and depression (Est = 2.95 [1.67–4.23]) were associated with a higher symptom burden. BMI (Est = −0.09 [−0.17 to −0.017]) and recent OI (Est = −0.270 [−0.501 to −0.040]) were associated with lower symptom burden (Figure 1c–g). Sex, chronic headaches, depression, BMI and OI accounted for 35.2% of variance in the data (RMSE = 5.80, AIC = 3343, *F* = 57.8, *p* < 0.001).

**TABLE 2:** Determinants of concussion symptom reporting.

A. Determinants of symptom reporting among non‐mTBI youths with an underlying medical condition					
Factor	Est	95% CI	β	*t*	*p*
Intercept	7.15	2.85–11.46		3.26	0.001
Age	−0.029	−0.25 – 0.19	−0.0096	−0.25	0.80
Sex (female)	1.27	0.18–2.36	0.17	2.29	0.022
BMI	−0.097	−0.17 to −0.017	−0.088	−2.38	0.017
Household size	0.36	−0.001 – 0.74	0.069	1.95	0.051
Parental education	−0.025	−1.07 – 1.02	−0.0035	−0.04	0.962
Prior TBI	0.14	−1.14 – 1.44	0.02	0.22	0.82
Sports participation	−0.37	−1.42 – 0.67	−0.051	−0.69	0.48
ADHD	0.40	−0.74 – 1.55	0.056	0.69	0.48
Anxiety	1.17	−0.05 – 2.40	0.16	1.87	0.061
Chronic headaches	7.05	5.93–8.17	0.97	12.35	<0.001
Depression	2.95	1.67–4.23	0.40	4.54	<0.001
Learning disability	1.06	−0.57 – 2.71	0.14	1.27	0.20
Orthopaedic injury	−2.09	−3.09 to −0.02	−0.28	−2.27	0.023

B. Determinants of temporal symptoms among youths with mTBI					
Factor	Est	95% CI	Partial η^2^	*t*	*p*
Intercept	0.227	0.025–0.430		2.208	0.028
Days since mTBI	−0.030	−0.033 to −0.026	0.486	−19.006	<0.001
Age	−0.030	−0.056 to −0.003	0.014	−2.235	0.026
Sex (female)	0.179	0.048 to 0.310	0.020	2.687	0.008
BMI	0.001	−0.010 to 0.012	7.40 × 10^−5^	0.175	0.86
Household size	0.096	0.063–0.129	0.070	5.672	<0.001
Parental education	−0.171	−0.283 to −0.059	0.016	−3.016	0.003
Prior TBI	0.165	0.027–0.293	0.015	2.374	0.018
Sports participation	−0.218	−0.365 to −0.071	0.022	−2.919	0.004
ADHD	−0.053	−0.243 to 0.136	7.67 × 10^−4^	−0.552	0.581
Anxiety	0.203	0.040–0.366	0.015	2.447	0.015
Chronic headaches	0.183	0.002–0.365	0.010	1.994	0.047
Depression	−0.081	−0.281 to 0.118	0.0013	−0.798	0.42
Learning disability	−0.039	−0.259 to 0.181	3.21 × 10^−4^	−0.349	0.72
Orthopaedic injury	0.150	−0.179 to 0.481	0.0024	0.897	0.371

*Note*: A: Results based on multivariate linear regression analysis (*n* = 543). Model characteristics: Adj *R* ^2^ = 0.355, RMSE = 5.74, AIC = 3348, *F* = 23.2, *p* < 0.001. Assumption checks: Durbin–Watson = 1.93 (*p* = 0.314), Goldfeld–Quandt = 1.11 (*p* = 0.21), Kolmogorov–Smirnov = 0.037 (*p* = 0.46). A reduced model using only significant variables (i.e. age, sex, sports participation, headaches, depression and orthopaedic injury) displayed the following characteristics: Adj. *R* ^2^ = 0.339, RMSE = 0.809, AIC = 1795, *F* = 64.0, *p* < 0.001. B: Results based on linear mixed model fit by REML (Conditional *R* ^2^ = 0.571, Marginal *R* ^2^ = 0.317, AIC = 2215, LRT *X* ^2^ = 449.6, *p* < 0.001). 998 obs, 380 groups; A reduced model using only significant variables (i.e. age, sex, household size, parent education, prior mTBI, sports participation, anxiety and chronic headache) displayed the following characteristics: Conditional *R* ^2^ = 0.569, Marginal *R* ^2^ = 0.316, AIC = 2188, LRT *X* ^2^ = 447.9, *p* < 0.001.

### Longitudinal symptom reports among youths with mTBI

**FIGURE 2:**
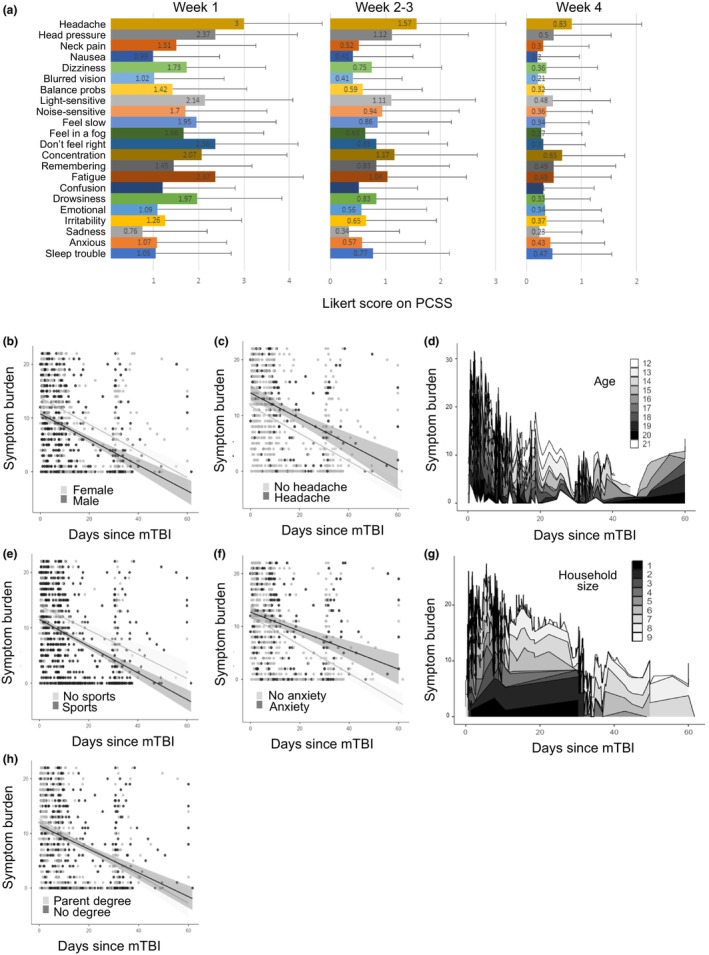
Symptom reports among youths with mild traumatic brain injury (mTBI). The bar plots display mean symptom scores (0–6 Likert scale) for 22 individual symptoms on the Post‐Concussion Symptom Scale (PCSS), as reported by 380 youths with mTBI within 1 week of injury, 2–3 weeks after injury and 4 weeks after injury (a). A multivariate mixed model fit by restricted maximum likelihood showed that longitudinal symptom burden was associated with sex (b), history of chronic headache (c), age (d), sports participation (e), history of anxiety (f), household size (g) and parent education (h). The scatterplots display symptom burden relative to days since mild traumatic brain injury (mTBI). Regression lines with 95% confidence intervals (CI) are split by dichotomous variables (sex, headache, sports participation, anxiety, parent education (presence/absence of 4‐year college diploma)). The area plots display 95% CI for symptom burden across days since mTBI for ordinal variables (age, household size).

Youths with mTBI (*n* = 380) completed 380 symptom surveys at enrolment (2 ± 2 days post‐mTBI), 351 surveys at the 1–2‐week follow‐up (351/380, 92.3%), and 258 at the 30‐day follow‐up (258/380, 67.9%). Over half of youths with mTBI (208/380; 54.7%) provided a retrospective estimate of their pre‐injury baseline symptom. At the time of enrolment, the median symptom burden among youths with mTBI was 13 (range = 0–22, IRQ = 10). The median symptom severity was 31 (range = 0–124, IQR = 41). Mean Likert scores were highest for headache (Mean = 3.0, SEM = 0.08), pressure in head (Mean = 2.37, SEM = 0.09), fatigue (Mean = 2.37, SEM = 0.06), don't feel right (Mean = 2.36, SEM = 0.09) and sensitivity to light (Mean = 2.14, SEM = 0.09). (Figure 2a). Median symptom burden declined at the 1–2‐week follow‐up (7, range = 0–22, IQR = 13, *t* = −10.3, *p* < 0.001) and the 30‐day follow‐up (1, range = 0–22, IQR = 7, *t* = −17.2, *p* < 0.001), relative to enrolment. Median symptom severity at 1–2 weeks (17, range = 0–104, IQR = 27, *t* = −10.9, *p* < 0.001) and 30 days (9, range = 0–99, IQR = 9, *t* = −16.6, *p* < 0.001) was also lower than at enrolment. Symptoms with the highest mean severity rating at the 30‐day follow‐up (31 ± 3 days post‐mTBI) were headache (0.83, SEM = 0.07), difficulty concentrating (0.65, SEM = 0.07), difficulty remembering (0.49, SEM = 0.07), fatigue (0.49, SEM = 0.06) and trouble falling asleep (0.47, SEM = 0.06). Time since injury (in days) had the greatest effect on headache (X^2^ = 176, Z = −12.1, *p* < 0.001) and the least effect on feeling nervous or anxious (X^2^ = 9.42, Z = −2.90, *p* = 0.004).

### Factors associated with longitudinal symptom reports among youths with mTBI

A linear mixed model was used to examine how medical and sociodemographic factors impacted symptom burden over time following mTBI (Table 2b). The model accounted for 31.7% of variance in the data (AIC = 2215, *p* < 0.001). Similar to youths without mTBI, higher symptom burden in the 30 days following mTBI was associated with female sex (Est = 0.179 [0.048–0.310]) and chronic headaches (Est = 0.183 [0.002–0.365]), whereas lower symptom burden in the 30 days following mTBI was associated with older age (Est = −0.030 [−0.056 to −0.003]) and sports participation (Est = −0.218 [−0.365 to −0.071]) (Figure 2b–e). Factors associated with longitudinal symptom trajectory that were unique to the mTBI cohort included anxiety (Est = 0.203 [0.040–0.366]), household size (Est = 0.096 [0.063–0.129]) and parent education (Est = −0.171 [−0.283 to −0.059]). (Figure 2f–h).

### Defining PSaC

All PSaC determinations were made based on self‐reported symptoms on the PCSS at the 30‐day follow‐up. When PSaC was defined as symptom burden ≥ the 90th percentile symptom burden of healthy youths without mTBI (i.e. ≥ 13 symptoms), 38/258 (14.7%) youths with mTBI met PSaC criteria (Table 3). When PSaC was defined as symptom severity ≥ the 90th percentile symptom severity of healthy youths without mTBI (i.e. Likert score ≥ 21), 39/258 (15.1%) youths with mTBI met PSaC criteria. Defining PSaC as a change in symptom burden by ≥2 symptoms relative to retrospective pre‐injury ratings resulted in 39/208 (18.8%) youths with mTBI meeting PSaC criteria. Defining PSaC as a change in symptom severity by >3 points relative to retrospective pre‐injury ratings resulted in 35/208 (16.8%) youths with mTBI meeting PSaC criteria. Nearly half of youths with mTBI (84/201, 41.8%) reported feeling <99% of normal at the 30‐day follow‐up. Defining PSaC as feeling <90% of normal resulted in 49/258 (18.9%) youths with mTBI meeting PSaC criteria.

**TABLE 3:** PSaC rates with various classification methods.

Classification method	Criterion	PSaC rate, mTBI group, *n* (%)	Misclassification rate, healthy non‐mTBI, *n* (%)	Misclassification rate, non‐mTBI with underlying medical condition, *n* (%)
Symptom burden on Day 30	≥13	38/258 (14.7)	21/194 (10.8)	199/534 (37.2)
Symptom severity on Day 30	≥21	39/258 (15.1)	21/194 (10.8)	242/534 (45.3)
Baseline burden (Day 30 vs. retrospective baseline)	≥2	39/208 (18.8)	NA	NA
Baseline severity (Day 30 vs. retrospective baseline)	≥4	35/208 (16.8)	NA	NA
What % of normal do you feel? (Day 30)	<90%	49/258 (18.9)	42/194 (21.6)	275/543 (50.6)

*Note*: 258 of 380 youths with mTBI completed symptom ratings through Day 30. Of those, only 208 provided baseline symptom ratings at the time of injury.

### Comparison of PSaC classification methods

The five PSaC classification methods showed 84.2% overall agreement (Fleiss' *ĸ* = 0.688 (0.600–0.783), *z* = 31.9, *p* < 0.001, Kendall's *W* = 0.754). Defining PSaC based on ‘feeling < 90% of normal’ showed 81.4% agreement with the definition involving Day 30 symptom burden (210/258, *ĸ* = 0.476, *p* < 0.001), 82% agreement with Day 30 symptom severity (211/258, *ĸ* = 0.489, *p* < 0.001), 89% agreement with retrospective pre‐injury symptom burden (179/201, *ĸ* = 0.630) and 92% agreement with pre‐injury symptom severity (185/201, *ĸ* = 0.720).

## DISCUSSION

This study of 1947 concussion symptom surveys from 1117 youths adds to existing evidence that medical and sociodemographic characteristics influence concussion symptom reporting (Asken et al., 2017; Brown et al., 2015; Custer et al., 2016; Iverson et al., 2015; Mayer et al., 2024; Moser et al., 2019). Influential factors among youths without mTBI were sex, history of chronic headache, depression, BMI and OI. Medical and sociodemographic factors not only impacted symptom reports among youths without mTBI, but they also influenced the longitudinal burden of symptoms in the 30 days following mTBI. Among youths with mTBI, age, sex, sports participation, history of chronic headache, anxiety, parent education, prior mTBI and household size were associated with symptom burden over time. Failure to account for these factors when assessing patients in the post‐concussion period could impact determinations of PSaC.

This study also illustrates how PSaC classification criteria may impact PSaC determinations. Although PSaC rates were similar between techniques using population‐based cut‐offs and techniques correcting for individual baseline symptoms, over 15% of cases lacked agreement and a large proportion of non‐mTBI youths with underlying medical conditions were misclassified as having PSaC in the absence of a baseline comparison.

The non‐mTBI population in this study purposefully included youths with neuropsychiatric symptoms that mirror mTBI (e.g. ADHD, anxiety, chronic headache and depression). Enriching for neuropsychiatric conditions among the control group provided a unique opportunity to assess the association of these conditions with symptom reporting. The median number of symptoms on the PCSS varied considerably between healthy, non‐mTBI youths (one symptom) and non‐mTBI youths with an underlying medical condition (nine symptoms). Symptoms with the highest mean Likert score for non‐mTBI youths with an underlying medical condition (headache and difficulty concentrating) were similar to those of the mTBI group, but differed from healthy, non‐mTBI youths (trouble falling asleep, nervous/anxious).

Consistent with a prior study in high school athletes by Iverson et al. (Iverson et al., 2015), these results show that female sex, headache history and psychiatric history are associated with symptom burden among youths without mTBI. However, this study extends these findings to non‐athletes and younger participants. Including these groups is important, since prior studies have suggested that symptom trajectory after mTBI may be unique among athletic populations and influenced by age (Mayer et al., 2024; Morgan et al., 2015). When assessing younger children and non‐athletes, both of whom typically lack baseline symptom assessments, clinicians should consider that symptom reports in these groups may be elevated even in the absence of mTBI.

Among the 380 youths with mTBI, female sex, history of chronic headache, anxiety, prior mTBI and larger household size were associated with higher symptom burden over time, whereas older age, higher parent education and sports participation were associated with lower symptom burden. Notably, many of the same factors that influenced baseline symptom reporting among youths without mTBI exerted identical effects on symptom burden over time among youths with mTBI (i.e. age, sex, history of chronic headache and sports participation). This finding suggests that individual‐level factors are important during baseline testing, but also impact the trajectory of symptoms after injury. In particular, the protective effect of sports participation on symptom recovery reaffirms differences in how athletes and non‐athletes report symptoms (Cicero et al., 2023). Growing evidence has shown that aerobic exercise may improve headaches, sleep disorders and neuropsychiatric symptoms in teens (Bektaş et al., 2015; Lang et al., 2013; Recchia et al., 2023). The protective effects of sports participation on symptom trajectory may be explained by these physiologic benefits, in combination with the incentive to return to play.

The study also identifies two social determinants of health associated with symptom reporting: parent education and household size. Given the importance of family education and concussion recovery (Ponsford et al., 2019), it is unsurprising that education might also reduce symptom reporting over time. The link between household size and symptoms is less clear but could be related to an optimized environment for sleep, focus and family/parent support (Atif et al., 2022; Kita et al., 2020). These findings support the growing recognition that social determinants of health play an important role in shaping concussion symptoms (Taylor et al., 2024).

Thirty days after injury, rates of PSaC among youths with mTBI ranged from 14.7% to 18.9%, depending on the classification method. Methods that relied solely on Day 30 symptom burden yielded similar rates of PSaC as those that corrected for baseline symptoms, but misclassification rates for non‐mTBI youths with an underlying medical condition were high (37.2%). Similarly, a single question, ‘What percent of normal do you feel?’ yielded comparable PSaC rates (18.9%) when the threshold was set at <90% of normal, but this simplistic method was even more likely to misclassify non‐mTBI youths with an underlying medical condition.

The current results show that variations in PSaC definition impact PSaC classification rates and suggest population‐based cut‐offs may misclassify PSaC in youths with sociodemographic or medical factors that influence symptom reporting. A recent study by Mayer et al. (Mayer et al., 2020) examined symptom reports from 162 youths with mTBI and 117 healthy controls and found an impact of classification method on PSaC accuracy. Specifically, that study found that standardized change algorithms (e.g. reliable change indices) were superior to simple change approaches with respect to inter‐rater reliability and suggested that methods eschewing retrospective ratings or statistically accounting for retrospective bias provide the best approach for PSaC assessment. While recognizing the benefits of complex statistical approaches for measuring standardized symptom change, we also propose that a simplistic method using a single question (e.g. ‘What percent of normal do you feel’) may provide a rapid screening approach for PSaC in clinical settings. When a threshold of 90% is set for ‘What percent of normal do you feel?’, this criterion identifies similar rates of PSaC among youths with mTBI as other standard classification approaches. However, given the misclassification rate of this approach among youths without mTBI, it would be important to complete full symptom surveys in screen‐positive cases before making a diagnosis of PSaC.

The current study has several limitations. The study relied on a single symptom rating scale (PCSS) and defined PSaC at 30 days post‐mTBI. Symptom profiles at 2‐ or 3‐months post‐mTBI were not available for this retrospective analysis. Symptom recovery was not determined for a sizable proportion of youths with mTBI (122/380), which could introduce retention bias. In addition, youths without mTBI provided symptom estimates only once, preventing calculation of reliable change indices across mTBI and non‐mTBI groups. Overall, the cohort was predominantly white, English‐speaking, non‐Hispanic and suffered from sport‐related mTBI, which may impact generalizability. Although a significant number of medical, demographic and social variables were available for this cohort, missing data for specific variables, such as household size and parent education, could have impacted their associations with concussion‐related symptoms and should be validated in an external cohort. Finally, the study focused on youths ages 11–21 years with mild brain injury. Future studies examining younger children and youths with moderate–severe TBI are necessary.

## CONCLUSION

This study demonstrates that concussion‐like symptoms are common among youths with neuropsychiatric conditions in the absence of mTBI. Concussion symptom reports can be impacted by sociodemographic factors, including age, sex, sports participation, parent education and household size. In patients with mTBI, these characteristics can influence PSaC determinations, especially when using classification techniques that fail to account for baseline symptoms. Although robust techniques such as reliable change are ideal for PSaC classification, there may be a limited role for a simple, single‐item question (i.e. ‘What percent of normal do you feel?’) as a PSaC screening tool in youths recovering from mTBI.

## AUTHOR CONTRIBUTIONS

**S. D. Hicks:** Conceptualization; funding acquisition; writing – original draft; methodology; formal analysis; supervision. **B. A. Harding:** Investigation; data curation; writing – review and editing; project administration. **R. Olympia:** Investigation; supervision; writing – review and editing. **J. Loeffert:** Writing – review and editing; investigation; methodology. **C. Onks:** Investigation; writing – review and editing; methodology. **R. C. Mannix:** Writing – review and editing; methodology; supervision; investigation; conceptualization. **D. A. Levine:** Investigation; writing – review and editing; supervision. **K. O. Yeates:** Writing – original draft; formal analysis; methodology.

## CONFLICT OF INTEREST STATEMENT

S. D. Hicks has previously served as a chief medical officer and paid scientific advisory board member for Quadrant Biosciences, and is named as a co‐inventory on intellectual property involving the use of salivary RNA for identification of neurologic conditions, such as concussion. The other authors declare no conflicts of interest.

## Supporting information


Figure S1:

## Data Availability

The data that support the findings of this study are available from the corresponding author upon reasonable request.
