# Supplementary figures and images for: Individual characteristics associated with youth symptom reports and persisting symptoms after concussion
Source: J Neuropsychol. 2026 Jan 31;20(2):343–60. doi: 10.1111/jnp.70031 (PMC13250370; doi:10.1111/jnp.70031)

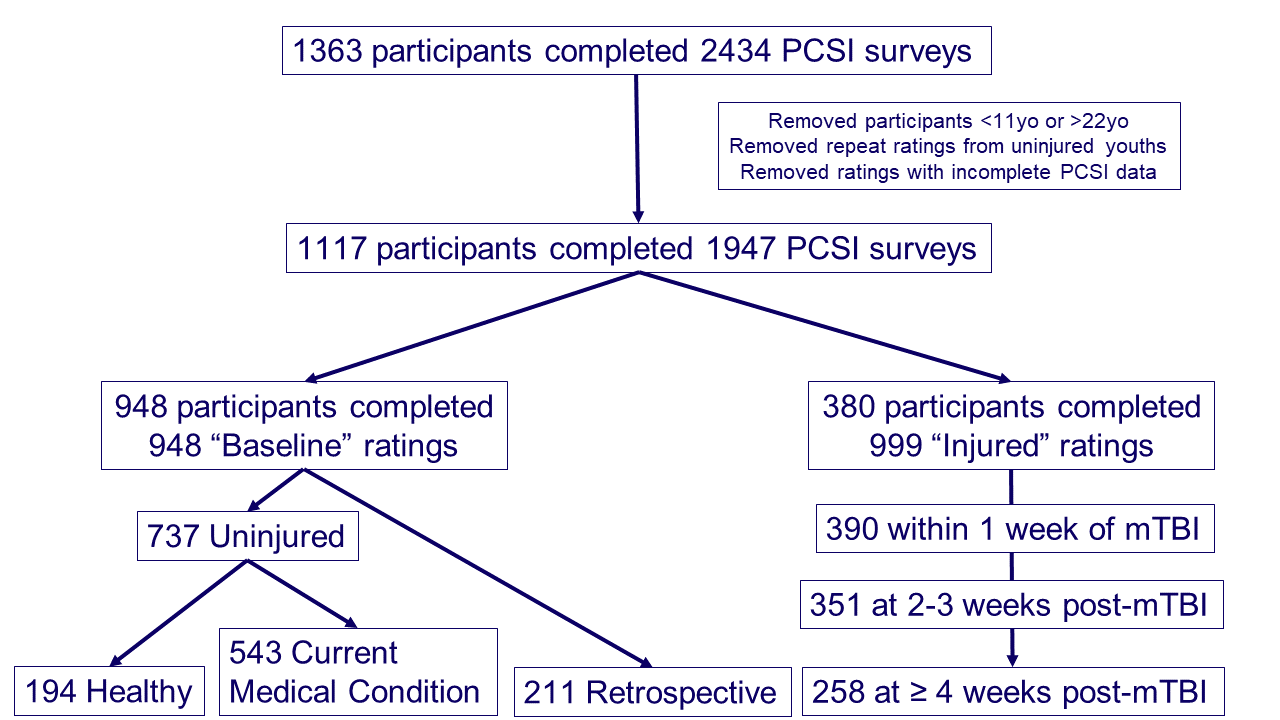

Supplement: Supplementary file 1 — Figure S1: [file JNP-20-343-s001.png]
